# How frail is frail in oncology studies? A scoping review

**DOI:** 10.1186/s12885-023-10933-z

**Published:** 2023-06-02

**Authors:** James A. Fletcher, Benignus Logan, Natasha Reid, Emily H. Gordon, Rahul Ladwa, Ruth E. Hubbard

**Affiliations:** 1 Division of Cancer Services, Princess Alexandra Hospital, 199 Ipswich Road, Woolloongabba, QLD 4102 Australia; 2 Faculty of Medicine, The University of Queensland, 199 Ipswich Road, Woolloongabba, QLD 4102 Australia; 3 Faculty of Medicine, Centre for Health Services Research, The University of Queensland, 199 Ipswich Road, Woolloongabba, QLD 4102 Australia

**Keywords:** Frailty, Frail elderly, Frailty index, Deficit accumulation, Geriatric assessment, Geriatric oncology

## Abstract

**Aims:**

The frailty index (FI) is one way in which frailty can be quantified. While it is measured as a continuous variable, various cut-off points have been used to categorise older adults as frail or non-frail, and these have largely been validated in the acute care or community settings for older adults without cancer. This review aimed to explore which FI categories have been applied to older adults with cancer and to determine why these categories were selected by study authors.

**Methods:**

This scoping review searched Medline, EMBASE, Cochrane, CINAHL, and Web of Science databases for studies which measured and categorised an FI in adults with cancer. Of the 1994 screened, 41 were eligible for inclusion. Data including oncological setting, FI categories, and the references or rationale for categorisation were extracted and analysed.

**Results:**

The FI score used to categorise participants as frail ranged from 0.06 to 0.35, with 0.35 being the most frequently used, followed by 0.25 and 0.20. The rationale for FI categories was provided in most studies but was not always relevant. Three of the included studies using an FI > 0.35 to define frailty were frequently referenced as the rationale for subsequent studies, however, the original rationale for this categorisation was unclear. Few studies sought to determine or validate optimum FI categorises in this population.

**Conclusion:**

There is significant variability in how studies have categorised the FI in older adults with cancer. An FI ≥ 0.35 to categorise frailty was used most frequently, however an FI in this range has often represented at least moderate to severe frailty in other highly-cited studies. These findings contrast with a scoping review of highly-cited studies categorising FI in older adults without cancer, where an FI ≥ 0.25 was most common. Maintaining the FI as a continuous variable is likely to be beneficial until further validation studies determine optimum FI categories in this population. Differences in how the FI has been categorised, and indeed how older adults have been labelled as ‘frail’, limits our ability to synthesise results and to understand the impact of frailty in cancer care.

**Supplementary Information:**

The online version contains supplementary material available at 10.1186/s12885-023-10933-z.

## Introduction

Frailty is a dynamic state of diminished physiological reserve and increased vulnerability to adverse events. It has been recognised as a prevalent and important consideration in the individualised management of older adults with cancer [1, 2]. Routine screening for geriatric conditions has been recommended for all adults over 65 or 70 years of age with a new cancer diagnosis [2–6]. In contrast to older adults in the community-dwelling or acute care settings, those living with cancer face the additional acute stressors of cancer symptoms and potential treatment-related toxicities. Frailty has significant implications for not only understanding the underlying health status of a potentially-vulnerable individual with cancer, but also in influencing oncological treatment decisions and discourse, and tailoring non-oncological interventions or supports. Clinicians try to determine those who are too frail for treatment, those who require modified treatment or additional supports, and those who are deemed fit for standard therapy. However, there is currently no consensus regarding the optimum frailty screening or measurement tool in this population.

The frailty index (FI) is one way in which frailty can be quantified [7]. The FI conceptualises frailty as a multi-dimensional risk state which can be measured by the number, rather than the nature, of health problems. An FI is calculated as a proportion of deficits using a well-defined method [8] e.g., someone with 6 deficits out of 40 counted has an FI of 0.15. As a continuous variable, ranging from zero (most robust) to a theoretical maximum of one (most frail), the FI affords great precision in risk stratification by capturing frailty gradations. In a scoping review of FI in the community and acute care settings, an FI ≥ 0.25 was the most frequently used score to diagnose people as frail, however this was used in less than half of the identified studies [7]. This score was derived from work by Rockwood and colleagues, which demonstrated that FI = 0.25 had construct and predictive validity to categorise community-dwelling adults as frail or non-frail [9, 10]. It correlated with the crossing point between robust and frail groups according to Fried et al.’s phenotype model of frailty [10], another well validated yet conceptually distinct definition of frailty in older persons [11, 12], and was predictive of institutionalisation and death. It also presented the crossing point between Clinical Frailty Scale (CFS) ‘apparently vulnerable’ (mean FI = 0.22) and ‘mildly frail’ (mean FI = 0.27) [10].

However, little is known regarding the validity of FI categories in the context of cancer, and variation in who is deemed frail may be used to determine trial eligibility or treatment allocation [13], and referral for additional assessments or supports [14, 15]. It is therefore important to understand how the FI has been categorised in oncology literature, and to understand the rationale for these decisions [16].

The objectives of this scoping review were: (i) to evaluate which FI categories (FI scores and labels) have been used in an oncology setting; and (ii) to identify why these categories were selected by the study authors.

## Methods

### Protocol and registration

The protocol for this scoping review protocol was prospectively registered with Open Science Framework (registration ID osf.io/gchq8) and developed in accordance with Preferred Reporting Items for Systematic Reviews and Meta-Analyses extension for Scoping Reviews criteria [17].

### Eligibility criteria

Articles were considered eligible for inclusion if they utilised a frailty index (FI) that met the criteria described by Searle and colleagues [18], and if the FI was categorised. There was no limitation to study design or year of publication, however only studies conducted in human adults with solid organ malignancies were included. Articles were excluded if they were not an original study or were only available as an abstract or protocol.

### Search strategy

A search of Medline, EMBASE, and Cochrane databases was conducted on 26 November 2021. Updated searches were performed to include CINAHL and Web of Science databases, as well as additional studies published before 22 July 2022 in all databases. Search results were imported into Covidence for screening, full text review, and data extraction. The full search strategy is available in the Supplementary appendix.

### Study selection

Two reviewers (JF and BL) independently performed the screening and full text reviews. Disagreements were resolved by consensus with a third reviewer (NR).

### Data extraction and analysis

Two reviewers (JF and BL) independently performed data extraction and disagreements were resolved by consensus with a third reviewer (NR). Extracted data included country, year of publication, study design, sample size, baseline demographics, and cancer-related details. FI data extracted included name, mean, categorised scores and labels, and justification for categorisation.

## Results

**Fig. 1:**
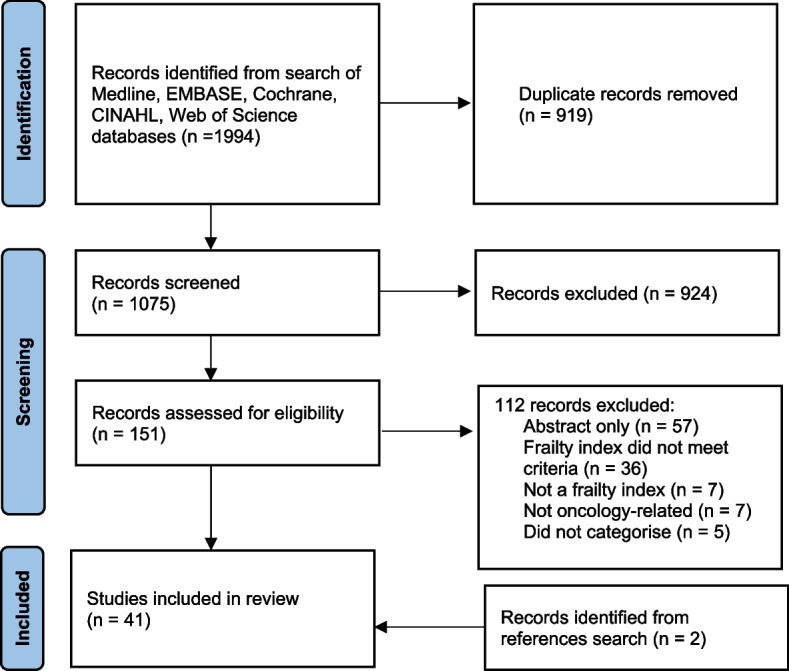
PRISMA diagram of search and study selection

The primary search yielded 1994 articles (Fig. 1). After removal of duplicates, abstract and full text screening, 41 studies were ultimately included.

### Study characteristics

All 41 studies [19–59] were published between 2014 and 2022, with the majority (*n* = 29, 71%) conducted in North America (Table 1). Thirty-six were of an observational study design, two studies reported secondary analyses of data from the same cluster randomised controlled trial, and three studies were non-randomised experimental trials. The median sample size was 541 (interquartile range [IQR] = 175–1136). The mean FI ranged from 0.05 to 0.31.

**Table 1:** Study characteristics and frailty index categories. Studies are listed in descending order according to the frailty index cut-off used to categorise older adults as frail, and then alphabetically by frailty index name

Author, Year, Country, Study Design	Cancer Type	N	Mean Age in Years (SD)	Frailty Index Name	FI Categories	FI Items	Mean FI (SD)	Frailty Prevalence	Rationale and Comments
**Giri 2021** **USA** [25] Observational	Gastrointestinal	455	Median 68 (IQR 64–74)	CARE Frailty Index	> 0.35 (frail) 0.2—0.35 (prefrail) < 0.2 (robust)	44	NR	36.8% frail 30% prefrail 33.2% robust	Referenced Searle et al. [27] which did not categorise FI
**Giri 2022** **USA** [26] Observational	Various	603	Median 69 (IQR 64–74)	CARE Frailty Index	> 0.35 (frail) 0.2—0.35 (prefrail) < 0.2 (robust)	44	NR	36.2% frail 29.0% pre-frail 33.2% robust	Referenced Searle et al. [27] which did not categorise FI
**Williams 2022** **USA** [39] Observational	Gastrointestinal	553	69.9 (7.1)	CARE Frailty Index	> 0.35 (frail) 0.20—0.35 (pre-frail) 0—0.20 (robust)	44	White median 0.3 (range 0.0–0.9) Black median 0.4 (range 0.0–0.8)	36.7% frail	Referenced Searle et al. [27] which did not categorise FI
**Guerard 2017** **USA** [27] Observational	Various including haematological	546	Median 72 (range 65–100)	Carolina Frailty Index (CFI)	> 0.35 (frail) 0.2—0.35 (pre-frail) 0—0.2 (robust)	36	NR	18% frail 24% pre-frail 58% robust	Nil
**Nishijima 2017** **USA** [33] Observational	Various	133	Median 74 (range 65–92)	Carolina Frailty Index (CFI)	≥ 0.35 (frail) 0.20 ≤ FI < 0.35 (pre-frail) < 0.20 (robust)	36	0.22 (0.16)	24% frail 22% prefrail 54% robust	Referenced study by co-authors Guerard et al. [25]
**Williams 2018** **USA** [37] Observational	Various including haematological	162	Median 71 (IQR 68–77)	Carolina Frailty Index (CFI)	> 0.35 (frail) 0.20—0.35 (pre-frail) < 0.20 (robust)	36	NR	21% frail 27% pre-frail 53% robust	Indirectly referenced work by co-authors Guerard et al. [18]
**Williams 2019** **USA** [38] Observational	Breast	63	70 (range 65–86)	Carolina Frailty Index (CFI)	> 0.35 (frail) 0.20—0.35 (pre-frail) < 0.20 (robust)	36	NR	5% frail 18% pre-frail 78% robust	Referenced two studies by co-authors Guerard et al. [27, 60]
**Zhou 2021** **USA** [58] Observational	Breast	46,027	Median 74 (NR)	Claims-based Frailty Index	≥ 0.35 (frail) 0.20—0.35 (pre-frail) ≤ 0.20 (fit)	93	NR	7.1% frail 36.7% pre-frail 66.6% fit	Referenced validation paper for claims-based frailty index [ref] and Cohen et al.[ref]
**Ahles 2021** **USA** [19] Observational	Breast	490^ꝉ^ including 162 non-cancer controls	72.6 (6.0)	Deficit Accumulation Frailty Index (DAFI)	≥ 0.35 (frail) 0.2 ≤ FI < 0.35 (prefrail) < 0.2 (robust/nonfrail)	44	NR	7% frail 24% prefrail 69% robust 7% missing	Referenced a study by a co-author, Cohen et al. [22]
**Ahles 2022** **USA** [20] Observational	Breast	490^a^ including 162 non-cancer controls	72.6 (6.0)	Deficit Accumulation Frailty Index (DAFI)	≥ 0.35 (frail) 0.2 ≤ FI < 0.35 (prefrail) < 0.2 (robust/nonfrail)	44	NR	7% frail 24% prefrail 69% robust 7% missing	Same cohort as Ahles 2021. [19] Indirectly referenced Cohen, [22] a study by a co-author
**Gilmore 2021** **USA** [23] Secondary analysis of RCT	Various including lymphoma	541^c^	76.6 (5.22)	Deficit Accumulation Index (DAI)	≥ 0.35 (frail) 0.2 ≤ FI < 0.35 (prefrail) < 0.2 (robust)	48	NR	32.5% frail 40.9% prefrail 26.4% robust	Referenced a study by a co-author, Cohen et al. [22]
**Gilmore 2022** **USA** [24] Secondary analysis of RCT	Various including lymphoma	541^c^	76.6 (range 70–96)	Deficit Accumulation Index (DAI)	≥ 0.35 (frail) 0.2 ≤ FI < 0.35 (prefrail) < 0.2 (robust	50	0.30 (0.15)	31% frail 41% prefrail 26% robust	Referenced a study by a co-author, Cohen et al. [22]
**Cohen 2016** **USA** [22] Observational	Various	500	73 (6.18)	Deficit-Accumulation Frailty Index (DAFI)	≥ 0.35 (frail) 0.2 ≤ FI < 0.35 (prefrail) < 0.2 (robust/nonfrail)	51	NR	11% frail 39% prefrail 50% nonfrail	Referenced a study by co-authors, Sheppard et al. [34] Also referenced Song et al. [61] and Theou et al., [62] which both used different FI categorisations
**Mandelblatt 2021** **USA** [31] Observational	Breast	708^b^ including 355 non-cancer controls	68.2 (6.0) cancer 67.9 (7.1) control	Deficits Accumulation Score	> 0.35 (frail) 0.20 ≤ FI < 0.35 (prefrail) < 0.20 (robust)	42	0.15 (0.08)	NR	Referenced Rockwood et al. [63] and Searle et al., [25] which did not categorise FI
**Bluethmann 2017** **USA** [21] Observational	Breast	990^b^	72.6 (5.9)	Frailty Index	> 0.35 (frail) 0.2 < FI ≤ 0.35 (pre-frail) 0–0.2 (robust)	35	NR	22.9% pre-frail/frail 77.1% robust	Referenced study by co-authors Sheppard et al. [34] Also referenced studies by Searle et al. [18] and Rockwood et al., [64] which did not report this FI categorisation
**Mandelblatt 2017** **USA** [29] Observational	Breast	1280^b^	72.4 (5.9)	Frailty Index	≥ 0.35 (frail) 0.2 ≤ FI < 0.35 (prefrail) < 0.20 (robust)	35	NR	5.1% frail 18.3% pre-frail 76.7% robust	Referenced a study by co-authors, Cohen et al. [22] Also referenced Rockwood et al. [64], Searle et al., [33] and three other studies which did not report this FI categorisation [65–67].
**Negrete-Najar 2021** **USA** [68] Observational	Pancreatic	440	Median 76 (range 70–91)	Frailty Index	≥ 0.35 (frail) 0.20 ≤ FI < 0.35 (pre-frail) < 0.20 (fit)	61	0.26 (0.09)	16.6% frail 58% prefrail 25.5% fit	Referenced study by Song et al., [61] which referenced Rockwood et al. [10]
**Weiss 2020** **USA** [36] Non-randomised experimental study	Lung	42	76.3 (range 71–84)	Frailty Index	> 0.35 (frail) 0.2 ≤ FI ≤ 0.35 (prefrail) < 0.2 (robust)	35	NR	42% frail 39% prefrail 19% robust	Nil
**Wang 2019** **China** [35] Observational	Lung	1020^d^	Median 65 (NR)	Frailty Index Based on Laboratory Variables (FI-LAB)	≥ 0.35 (frail) 0.20—0.35 (pre-frail) 0—0.2 (robust)	44	median 0.14 (range 0—0.61)	4.9% frail 26.4% pre-frail	Referenced Cohen et al. [22]
**Sheppard 2014** **USA** [34] Observational	Breast	1288^b^	72.78 (6.05)	Frailty Score	≥ 0.35 (frail) 0.2 ≤ FI < 0.35 (prefrail) < 0.2 (robust)	35	NR	4.9% frail 18.7% prefrail 76.4% robust	Referenced studies by Searle et al. [18] and Rockwood et al., [27] which did not report this FI categorisation
**Mandelblatt 2016** **USA** [28] Observational	Breast	1280^b^	72.7 (5.9)	Searle Index	≥ 0.35 (frail) 0.2 ≤ FI < 0.35 (prefrail) < 0.2 (robust)	35	NR	5.1% frail 18.3% pre-frail 76.7% robust	Referenced co-authored study by Sheppard et al. [34] Also referenced Rockwood et al., which did not report this FI categorisation. [64]
**Mandelblatt 2018** **USA** [30] Observational	Breast	691^b^ including 347 non-cancer control	67.8 (7.0) cancer 68.1 (6.1) control	Searle's Deficits Accumulation Index	≥ 0.35 (frail) 0.2 ≤ FI < 0.35 (prefrail) < 0.20 (robust)	40	NR	25.6% prefrail/frail 74.4% robust	Referenced Rockwood et al., [64] which did not report this FI categorisation
**Martinez-Tapia 2022** **France** [40] Observational	Various	1136	Median 80 (IQR 76–85)	Geriatric Assessment Frailty Index	≥ 0.30 (unfit) < 0.30 (fit)	52	NR	88.8% unfit 11.2% fit	Referenced Searle et al. [27] which did not categorise FI. Author comments that an FI > 0.20 would have categorised 99% as unfit and limited analysis
**Inci 2021** **Germany** [59] Observational	Ovarian	144	Median 58 (range 18–87)	Frailty Index	> 0.26 (frail) ≤ 0.26 (non-frail)	30	NR	33% frail 67% non-frail	Authors used receiver operator characteristic analyses and logistic regression to determine that an FI > 0.26 showed the best predictive threshold for severe complications (> IIIB according to Clavien Dindo) For overall survival the Log Rank test showed the best cut-off with FI > 0.15
**Giannotti 2022** **Italy** [43] Non-randomised experimental study	Gastrointestinal	208	Median 80 (IQR 77.4–84.0)	40-Item Frailty Index (40-FI)	≥ 0.25 (frail) 0.8 < FI < 0.25 (prefrail) ≤ 0.08 (fit)	40	Median 0.15 (IQR 0.10—0.26)	NR	Referenced an abstract which did not categorise frailty [69].
**McCarthy 2018** **Australia** [44] Observational	Solid tumour	175	72 (5.2)	FI-CGA	> 0.25 (frail) ≤ 0.25 (fit)	42	0.31 (0.14); 0.27 (0.21—0.39)	53.7% frail 46.3% fit	Referenced a review article by Rockwood et al. [70] Demonstrated construct validity against fitness and vulnerability as measured by the VES-13, and by doctor assessment
**Reiser 2021** **Austria** [46] Observational	Gynaecological	83	84.2 (3.5)	Frailty Index	≥ 0.25 (frail) < 0.25 (non-frail)	31	0.19 (0.16)	24.1% frail 75.9% non-frail	Referenced study by Song et al., [61] which referenced Rockwood et al. [10] The latter demonstrated the construct and predictive validity of FI > 0.25, which represented the crossing point between robust and frail groups measured using the phenotypic frailty model
**Giannotti 2019** **Italy** [42] Non-randomised experimental study	Gastrointestinal	99	80.18 (5.88)	Frailty Index (FI)	≥ 0.25 (frail) 0.8 < FI < 0.25 (prefrail) ≤ 0.08 (fit)	40	0.22 (0.13)	40.5% frail 50.5% prefrail 9% fit	Referenced Rockwood et al.’s study, [71] which demonstrated construct and predictive validity of CFS categories. FI 0.25 represented the crossing point between CFS 4 (‘apparently vulnerable’, mean FI 0.22), and CFS 5 (‘mildly frail’, mean FI 0.27) Authors conducted pair-wise analyses of ROC curves for CGA and FI that showed similar accuracy in identifying 1-year mortality and functional outcomes. An FI cut-off of 0.19 showed the best predictive threshold for 1-year mortality, and between 0.15 and 0.18 for 1-year functional status
**Pérez-Zepeda 2016** **Mexico** [45] Observational	Various including non-cancer population	8 022: 288 with cancer	70.6 (7.4)	Frailty Index (FI)	≥ 0.25 (frail)	55	0.196 (0.108)	29.9% frail	Referenced Rockwood et al. [10]
**Geessink 2017** **Netherlands** [41] Observational	Various including non-cancer population	7 493: 751 with cancer	79.1 (6.5)	TOPICS-FI38	> 0.25 (frail)	38	0.23 (0.13)	NR	Nil
**Zhang 2022** **USA** [47] Observational	Various	2 050 cancer survivors including 9 474 controls	Cancer survivors, 72.6 (7.1)	Frailty Index (FI)	> 0.21 (frailty) 0.10 < FI ≤ 0.21 (prefrailty) ≤ 0.10 (fitness)	45	NR	55.9% frail 38.2% prefrail 5.9% fit	Referenced four studies validating an FI ≥ 0.21 in the National Health and Nutrition Examination Survey [72–75].
**Bensken 2022** **USA** [48] Observational	Breast Colorectal Prostate Lung	29 140	NR	Claims Frailty Index (CFI)	≥ 0.4 (severely frail) 0.3 < FI < 0.4 (moderately frail) 0.20 < FI < 0.30 (mildly frail) 0.10 < FI < 0.20 (pre-frail) < 0.10 (non-frail)	93	Breast 0.15 (0.06) Colorectal 0.16 (0.06) Lung 0.16 (0.07) Prostate 0.13 (0.05)	3.5% severely / moderately frail 14.2% mildly frail 75.4% pre-frail 7% non-frail	Referenced CFI validation study by a co-author, [76] which did not categorise the CFI, but did demonstrate that 0.1 increments predicted increased risk of mortality, functional decline, mobility impairment and recurrent falls
**Cooper 2022** **USA** [50] Observational	Lung	73	Median 76.7 (IQR 72.3 – 80.5)	Comprehensive Geriatric Assessment-Based Frailty Index (FI-CGA)	> 0.2 (frail) > 0.4 (severe frailty) 0.2 < FI ≤ 0.4 (occult frailty) ≤ 0.2 (non-frail)	45	NR	38.3% frail 6.8% severe frailty 31.5% occult frailty 61.6% non-frail	Referenced Rockwood et al. [77] ‘Occult frailty’ was referred to as a level of frailty often missed by surgical teams without the use of CGA
**Shen 2021** **China** [52] Observational	Lung	997^d^	66.07 (4.90)	Electronic Frailty Index (EFI)	≥ 0.20 (frail) < 0.20 (non-frail/robust)	35	NR	19.7% frail 80.3% non-frail/robust	Referenced Cohen et al., [22] and a study which evaluated a modified frailty index (mFI) [78].
**Tariciotti 2022** **Italy** [53] Observational	Meningioma	165	Median 63 (IQR 52–72)	Frailty Index (FI)	> 0.20 (frail) 0.10—0.20 (semi-fit) < 0.10 (fit)	34	Median 0.16 (IQR 0.06–0.18)	11.5% frail 46.7% semi-fit 41.8% fit	Referenced Searle et al. and Mitnitski et al., neither of which categorised frailty. [18, 79]
**Hembree 2021** **USA** [51] Observational	Various	189	Median 62.0 (range 26–87)	Frailty Index (FI) And Test Based Frailty Index (TBFI)	> 0.4 (severely frail) 0.3—0.4 (moderately frail) 0.2—0.3 (mildly frail) 0—0.2 (non-frail)	53	0.28 (0.12)	20.1% severely frail 30.7% moderately frail 32.8% mildly frail 10.5% non-frail	Referenced Jayanama et al. [80]
**Cheng 2022** **USA** [49] Observational	Non-small cell lung cancer	42 204	74.1 (6.3)	Veterans Affairs Frailty Index (VAFI)	> 0.3 (moderate-to-severely frail) 0.2—0.3 (mildly frail) 0.1—0.2 (pre-frail) 0—0.1 (non-frail)	31	0.25 (0.13)	27.8% moderate-severely frail 27.8% mildly frail 31.6% pre-frail 12.9% non-frail	Referenced three studies validating FI ≥ 0.21, with slightly different categories and labels. [72, 81, 82]
**Narasimhulu** **2020** **USA** [55] Observational	Ovarian	169^e^	Frail: 67.9 (9.4) Non-frail: 62.3 (10.7)	Frailty Deficit Index	≥ 0.15 (frail) < 0.15 (non-frail)	30	NR	17.2% frail 82.7% non-frail	Referenced authors’ prior studies in the same population [54, 56]
**Kumar 2017** **USA** [54] Observational	Ovarian	535^e^	64.3 (11.3)	Frailty Deficit Index (FI)	≥ 0.15 (frail) < 0.15 (non-frail)	30	median 0.08 (IQR 0.03—0.14)	24.% frail 75.5% non-frail	Authors derived the cut-off that yielded the highest Youden's index for their three binary outcomes (Accordion Grade 3 or 4 complication, 90-day mortality, receipt of chemotherapy within 42 days), while yielding the highest separation in outcome rates between frail and non-frail
**Yao 2019** **USA** [56] Observational	Ovarian	535^e^	64.3 (11.3)	Frailty Index	≥ 0.15 (frail) < 0.15 (non-frail)	30	NR	24.5% frail 75.7% non-frail	Same group and study population as Kumar et al. [54]
**Jauhari 2020** **UK** [57] Observational	Breast	67 925 External validation 4230	NA	Secondary Care Administrative Records Frailty (SCARF) Index	≥ 0.19 (severe frailty) 0.12—0.18 (moderate frailty) 0.06—0.11 (mild frailty) ≤ 0.05 (fit)	36	0.05 (NR)	3.3% severe frailty 6.6% moderate frailty 11% mild frailty 79.2% fit	No reference for categories Authors tested internal validation, and then external validation in a separate cohort of 4230 women

*Abbreviations: FI* Frailty index, *SD* Standard deviation, *IQR* Interquartile range, *NR* Not reported

^a^, ^b^, ^c^, ^d^,^e^ studies with same cohort, or subgroups of the same cohort of participants

Fifteen studies were specifically conducted in the medical oncology setting [21, 22, 25, 26, 28–31, 34–36, 39, 44, 52, 55], ten studies in surgical oncology [42, 43, 46, 50, 53, 54, 56–59], and the remainder were either mixed or not specified [19, 20, 23, 24, 27, 32, 33, 37, 38, 40, 41, 45, 47–49, 51]. Fourteen studies included a range of cancers and the rest focused on individual cancer types [22–27, 32, 33, 37, 40, 41, 45, 47, 48, 51]. There were 11 studies of breast cancer [19–21, 28–31, 34, 38, 57, 59], of which six studies were different secondary analyses of the same prospective clinical trial [21, 28–31, 34]. Similarly, two [35, 52] of the five lung cancer studies utilised the same retrospective database [35, 36, 49, 50, 52], as did three [54–56] of five studies of gynaecological cancers [46, 54–56, 58]. Four studies evaluated gastrointestinal cancers [25, 39, 42, 43], one studied pancreatic cancer [32], and one specifically evaluated participants with meningiomas [53].

### Frailty index categories

**Fig. 2:**
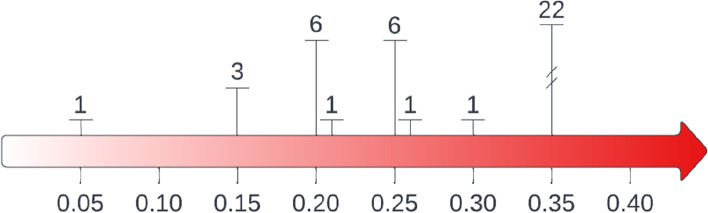
Distribution of frailty index cut-off points used to define frailty. The frailty index is a continuous measure between zero (least frail) and one (most frail). Here, the number of studies using different cut-off points to categorise adults with cancer as frail are highlighted. Significant variation in these frailty index cut-off points is evident

In 22 studies (54%) an FI ≥ 0.35 was used to categorise people as frail (Fig. 2) and an FI between 0.20 and 0.35 categorised people as prefrail [19–39]. Fourteen of the 22 studies referenced one of three oncology studies as rationale for their FI categorisation [22, 27, 34]. Sheppard et al., [34] published in 2014, referenced work which did not use similar FI categorisation. Sheppard et al. reported that prefrailty/frailty (FI ≥ 0.20) predicted treatment non-initiation in women with breast cancer. Cohen et al., [22] published 4 years later, referenced Sheppard et al. and reported that frailty predicted an increased likelihood of hospitalisation and treatment discontinuation in older adults commencing chemotherapy. Further, they determined the optimal FI cut-off for their individual outcomes was comparable to FI = 0.20. Guerard et al., [27] published in 2017, did not provide a rationale for their categorisation, however reported that frailty (defined as FI > 0.35) predicted an increased likelihood of all-cause and cancer-specific mortality in a heterogenous population of older adults with cancer, thereby establishing the predictive validity of their frailty categorisation.

An FI ≥ 0.25 was the next most commonly used cut-off to categorise frailty in six (15%) studies [41–46]. Four of these studies either directly, or indirectly, referenced validation studies by Rockwood and colleagues [42, 44–46]. One of these reported predictive validity against comprehensive geriatric assessment [42], and another validated their FI with respect to Vulnerable Elderly Survey (VES-13) scores and treatment completion outcomes [44]. Six studies defined frailty as an FI ≥ 0.20 [47–52], one used FI > 0.21 [47], and one additional study arbitrarily defined an FI > 0.30 as ‘unfit’, commenting that using an FI > 0.20 would have categorised 99% of their population as ‘unfit’ and limited their statistical analysis [40]. Four of these studies defined categories of increasing frailty, most often with 0.1 increments in FI [48–51]. References for these studies varied, including work by Rockwood and colleagues [76, 80].

Two groups defined the optimum FI cut-off point for predicting adverse outcomes in women with ovarian cancer [54–56, 59]. The first [54–56] reported that an FI = 0.15 was the cut-off that yielded the highest Youden's index for their three binary outcomes (Accordion Grade 3 or 4 complication, 90-day mortality, receipt of chemotherapy within 42 days), while yielding the highest separation in outcome rates between frail and non-frail. Using similar methodology, another group [59] reported that FI > 0.26 showed the best discriminative ability for severe post-operative complications (≥ IIIb by Clavien-Dindo criteria) in a younger cohort of women with predominantly advanced ovarian cancer. An FI > 0.15 was determined to be the best cut-off for overall survival in this population.

The final study arbitrarily defined frailty as FI ≥ 0.06, and further divided this into mild frailty (FI = 0.06 – 0.11), moderate frailty (FI = 0.12 – 0.18) and severe frailty (FI ≥ 0.19) [57]. Internal validity was tested in a large cohort of women with breast cancer, and external validity in a second large representative cohort [57].

## Discussion

This scoping review demonstrated significant variability in FI categorisation in older adults with cancer. An FI ≥ 0.35 was the most frequently used cut-off point to categorise frailty, followed by an FI ≥ 0.25. While most authors provided a rationale for their categorisation, many of the cited studies were not relevant, and the most frequently referenced oncological studies of FI in this context did not clearly justify or validate their cut-off points. Across treatment contexts, few studies sought to demonstrate construct validity or to establish optimal FI categories [22, 42, 51, 54, 57, 59].

This is the first review to evaluate FI categorisation in older adults with cancer. Significant variability in FI categorisation has also been reported in a recent review of the most highly-cited studies in older adults in the community, acute care, and residential care settings [7]. In contrast with this prior review, where an FI ≥ 0.25 was common, a significantly higher FI (≥ 0.35) was most frequently used to define frailty in oncology studies.

While the three most frequently cited oncology reference studies demonstrated associations between pre-frailty/frailty and adverse outcomes, the rationale for arriving at this FI categorisation was not clear [22, 27, 34]. Further, the nomenclature of ‘pre-frailty’ in this context would be more congruent with the measurement of frailty using the Fried Frailty Phenotype, which conceptualises frailty as a syndrome with three categories of fit, pre-frail, and frail. This is in contrast to the deficit accumulation model which considers frailty along a continuum, in which gradations of severity can be appreciated [18, 83]. It is also interesting to note that of these three studies, one collapsed ‘prefrailty’ (FI ≥ 0.20) and ‘frailty’ (FI ≥ 0.35) into a single category for their statistical analysis [34], and another identified an optimum FI cut-off point approximating their level of prefrailty (FI ≥ 0.20) [22]. FI cut-off points between 0.21 and 0.25 were used to define frailty, rather than ‘prefrailty’, in a further seven studies.

The predictive validity of these cut-off points (FI > 0.21, > 0.25, > 0.35) have been tested in community-dwelling adults by Hoover et al. [84], who reported four frailty categories for hospital-related outcomes (non-frail FI < 0.1, pre-frail 0.1 < FI ≤ 0.21, frail FI > 0.21, and most frail FI ≥ 0.45). These cut-offs also correspond well with the mean FI for increasing levels of the Clinical Frailty Scale (CFS): very fit (CFS 1, mean FI = 0.09), apparently vulnerable (CFS 4, mean FI = 0.22), and severely frail (CFS 7, mean FI = 0.43) [71].

Variability in the categorisation of frailty contributes to inconsistency in understanding the true impact of frailty on outcomes in older adults with cancer [2, 7]. These disparities should be taken into account when interpreting the data, as one patient may be categorised as robust in one study, and frail in another. Much like chemotherapy toxicity calculators [85], the FI is intended to inform intrinsic vulnerability and risk, rather than to discriminate between treatment options. Maintaining the FI as a continuous variable can be advantageous to mitigate this and to understand the associations between frailty and outcomes, however the importance of validated categorisations must also be acknowledged. Given the previously discussed findings, it could be suggested that an FI greater than 0.20 or 0.25 may be most appropriate to identify those who are at increased risk of adverse events and to categorise this group as frail. It is likely that studies defining frailty with an FI ≥ 0.35 captured a significantly more frail, and therefore more vulnerable population than studies using other validated cut-offs [71]. Graded frailty severity (e.g., mild, moderate, severe), reported in only five of the included oncology studies, may be a more useful method to assist researchers and clinicians. This would parallel chemotherapy toxicity calculators [85, 86], however more research is required to determine optimum FI cut-offs to discriminate between outcomes, and this may vary across tumour streams [42, 54].

Despite a number of studies including some patients with lymphomas, a limitation to this review was the exclusion of studies evaluating solely haematological malignancies. While the aim of this review was to determine FI categories and their rationale, the lack of validated FI categories in the present review means that these findings cannot likely be extrapolated to other haematological populations.

## Conclusion

This scoping review demonstrated variability in how oncological studies categorise frailty in older adults with cancer. While some studies sought to determine optimal cut-off points to define frailty in specific populations, FI categories were otherwise not well validated in the general oncology setting. Further work is therefore required to validate frailty categories in this context, and at the present time, the FI may be best reported as a continuous variable to understand an older adult’s level of frailty.

## Supplementary Information

**Additional file 1.**

## Data Availability

All data generated or analysed during this study are included in this published article.
