# Supplementary material for: How frail is frail in oncology studies? A scoping review
Source: BMC Cancer. 2023 Jun 2;23:498. doi: 10.1186/s12885-023-10933-z (PMC10236730; doi:10.1186/s12885-023-10933-z)
Supplement: Supplementary file 1 — Additional file 1. [file 12885_2023_10933_MOESM1_ESM.docx]

Appendix

Search Terms

Pubmed:

("frailty index" OR "rockwood frailty" OR "deficit accumulation" OR "accumulation of deficits") AND (neoplasms[Mesh] or cancer[TW] or malignan*[TW] or neoplas*[TW] or tumour*[TW] or tumor*[TW] or carcinoma*[TW])

EMBASE:

('frailty index' OR 'rockwood frailty' OR 'deficit accumulation' OR 'accumulation of deficits') AND ('cancer*':ti,ab,kw OR 'neoplasm':ti,ab,kw OR 'malignan*':ti,ab,kw OR 'tumour*':ti,ab,kw OR 'tumor*':ti,ab,kw OR ‘carcinoma*’:ti,ab,kw)

Cochrane:

#1 ("frailty index" OR "rockwood frailty" OR "deficit accumulation" OR "accumulation of deficits")

#2 neoplasms[MeSH]

#3 (cancer* or neoplasm* or malignan* or tumor* or tumour* or carcinoma*):ti,ab,kw

#1 AND (#2 or #3)

CINAHL

TX ( "frailty index" OR "rockwood frailty" OR "deficit accumulation" OR "accumulation of deficits" ) AND (MM ( neoplasms or cancer ) OR TX ( cancer* or neoplasm* or malignan* or tumor* or tumour* or carcinoma*) )

Filters: Human; end date July 2022

Web of science

("frailty index" OR "rockwood frailty" OR "deficit accumulation" OR "accumulation of deficits") AND (cancer* or malignan* or neoplas* or tumour* or tumor* or carcinoma*)

Filter: end date 22 July 2022
